# Addressing health information inequities: making evidence-based clinical content more accessible in low- and middle-income primary care

**DOI:** 10.1136/bmjgh-2023-013814

**Published:** 2025-12-19

**Authors:** Ruth Vania Cornick, Sandy Claire Picken, Ajibola Awotiwon, Mareike Rabe, Camilla Wattrus, Tasneem Fredericks, Venessa Timmerman, Lara R Fairall

**Affiliations:** 1 Knowledge Translation Unit, University of Cape Town Lung Institute, Cape Town, Western Cape, South Africa; 2 Knowledge Translation Unit, University of Cape Town Faculty of Health Sciences, Observatory, South Africa; 3 University of Cape Town, Rondebosch, South Africa; 4 knowledge translation unit, University of Cape Town, Rondebosch, South Africa; 5 School of Life Course & Population Sciences, King’s College London, London, UK

**Keywords:** Decision Making, Global Health, Health systems, Delivery of Health Care

## Abstract

Disparity in access to trustworthy health information between high-income and low-income settings remains stark and contributes to global health inequity. The volume of new clinical practice guidelines a healthcare provider needs to digest to deliver up-to-date, evidence-based care is overwhelming, particularly in primary care, where the scope is comprehensive. However, many low- and middle-income countries (LMICs) lack the resources to tailor guidance for their realities. International standards for adaptation or adoption of existing guidelines tend to focus on a single clinical topic and still require considerable evidence synthesis expertise, slowing provision of up-to-date, relevant protocols for the primary care provider.

The Practical Approach to Care Kit (PACK) guide covers most conditions managed in primary care. It has been introduced to South Africa, Ethiopia, Brazil, Nigeria, Botswana and Indonesia to support primary care reforms. This paper describes the reference repository and updating mechanisms underpinning the PACK Global guide (that forms a template for local adaptation) so that it reflects latest international evidence and WHO guidance. The referencing and updating mechanism to curate its 3689 recommendations drew on the established evidence synthesis processes of the British Medical Journal’s Best Practice and the WHO. The challenges of maintaining this content set were largely funding and resource constraints in our small team. We are exploring how advances in generative artificial intelligence might expedite review of the large clinical guidelines and policies required for PACK updates as well as address limitations of current database software as a content management system, to facilitate editorial and publication processes.

Leveraging existing evidence synthesis processes appears to be a feasible approach to maintaining a comprehensive LMIC primary care clinical content set and may go some way to improving access to up-to-date health information, thus addressing global health inequities.

**Summary box**

- Global health inequities are exacerbated by disparities in access to up-to-date clinical information.
- International standards for the adoption and adaption of clinical guidance tend to focus on a single topic and still require scarce evidence synthesis expertise.
- The Practical Approach to Care Kit (PACK) guide has been introduced to several low- and middle-income countries to support comprehensive primary care delivery.
- It leverages existing evidence synthesis processes and expertise of the British Medical Journal’s Best Practice and the WHO to update its 3689 clinical recommendations.
- The number and length of WHO guidance documents slow down the updating process. We are exploring advances in generative artificial intelligence for mechanisms to expedite PACK content review.

## Introduction

 The volume of new knowledge a primary care clinician should digest to remain up-to-date and deliver evidence-based care is overwhelming. A 2004 study estimated it would take 627.5 hours per month to read new primary care publications.^1^ This volume has grown exponentially—a PubMed search for ‘diabetes’ produced 23 828 articles for the year 2000, 43 569 for 2010 and 73 900 for 2020.^2^

Clinical evidence is most readily available to clinicians as regularly updated clinical practice guidelines and electronic clinical decision support (CDS) products.^3^ However, disparity in access to health information between high- and low-income settings remains stark, contributing to global health inequity.^4^ Subscription-based and high-income country focused, electronic CDS products are largely inaccessible to low- and middle-income country (LMIC) primary care clinicians. WHO clinical guidelines form the standard of care for LMIC clinical decision-making. However, many LMIC ministries of health lack time, funds and expertise to regularly adjust the plethora of lengthy WHO guidelines for local realities.^5^ Standards for adaptation or adoption of clinical practice guidelines from elsewhere tend to focus on single clinical topics,^6^ inadequate for comprehensive primary care.

### How can clinical guidance be made accessible to LMIC primary care providers that is based on the latest evidence and relevant to their setting?

The Practical Approach to Care Kit (PACK) guide has been adopted by government health departments in South Africa,^7^ Botswana,^8^ Brazil,^9^ Indonesia and Ethiopia^10^ and piloted in Nigeria^11^ to support primary healthcare reforms and make evidence and policy accessible at point of care. 34 localisations or updates of the guide have been published in hard and soft copy over 20 years, making it one of the most updated go-to resources for LMIC health workers.^12^

A training programme that aims to embed PACK’s use in primary care consultations^13 14^ has reached nearly 300 000 primary care clinicians in almost 13 000 healthcare facilities. A localisation package mentors its in-country adaptation to local policy, resources and epidemiology, implementation and evaluation.^15^

The guide’s 150-page content covers over 500 symptoms, diagnoses and chronic conditions, including communicable diseases, non-communicable diseases, mental illness, women’s health and palliative care. Its comprehensive scope speaks to the need of patient, provider and policy-maker, supporting a range of impacts in primary care delivery. These include improving clinical outcomes,^16 17^ responding to infectious disease outbreaks,^18^ streamlining multimorbidity care^19^ and reconciling pharmacy stocks with local medicines lists.^20^ The version for adult care is most widely used, with child^21^ and adolescent versions undergoing early implementation and evaluation.^22 23^ Latest versions are available for download: https://knowledgetranslation.co.za/downloads/

Underpinning this content set is a separate reference repository that contains the evidence for each of the PACK guide’s recommendations. Used by the PACK editorial team and in-country localisers, it supports the guide’s update, expansion and in-country localisation.

This practice paper forms part of a collection describing PACK’s role supporting LMIC health system reforms. It focuses on PACK’s reference repository and updating mechanisms, the challenges of maintaining these, lessons learnt and plans for further development. It may interest global health practitioners, policymakers, evidence synthesis experts and guideline developers exploring ways to make clinical information more accessible to LMIC healthcare practitioners.

### PACK guide development

Work on the guide was led by the PACK editorial team: doctors with clinical primary care, medical editing and evidence synthesis experience. All are co-authors or acknowledged at the end of this paper. The PACK guide was first developed in a specific primary care context for the Western Cape Province, South Africa. Development drew on developer clinical experience, disease burden^24^ and policy in local primary care. Informed by clinical guidance development standards, we engaged stakeholders—policy-makers, health system managers and end-user primary care clinicians (nurses and doctors)—to ensure the guide was acceptable and feasible for everyday use and addressed common presentations to primary care, including symptoms, even if evidence was sparse.^25^ The guide’s syndromic, comprehensive approach demanded interrogation of the relevance and practical applications of policy. Where local policy gaps or discrepancies made for confusing or impractical clinical recommendations, we consulted WHO guidance, sentinel guidelines, systematic reviews and primary studies to ensure a clinically coherent approach.

For PACK to form a template for in-country localisation by policy-makers and technical support partners to local policy, disease burden and resource constraints in other LMICs, it needed to reflect ‘global’ not local sources.^1^ We explored various methodologies for the process to create ‘PACK Global’ from PACK Western Cape. These are summarised in table 1.

**Table 1:** Summary of methodologies for deriving the ‘global’ evidence sources for PACK recommendations

	Advantage	Disadvantage
PICOT	Rigorous evaluation of evidence base for a recommendation	Time-consuming Difficult to scale across thousands of recommendations Not useful for all types of recommendation No streamlined mechanism for PACK updates
GRADE	Rigorous evaluation of evidence base for a recommendation	Time-consuming Difficult to scale across thousands of recommendations Not useful for all types of recommendation No streamlined mechanism for PACK updates Need to be applied in context.
Leveraging Best Practice processes	Established robust systematic mechanisms for maintaining content Content covers a wide range of conditions. Provided mechanism for PACK updates. Freely accessible to us through a partnership with BMJ	High-income country focus Little guidance or ‘suggested approach’ for scenarios where there is limited evidence
Leveraging WHO processes	More focus on LMIC epidemiology and resource constraints Content freely available online	Some WHO guidance is out of date. Gaps in guidance for some conditions

BMJ, British Medical Journal; LMIC, low- and middle-income country; PACK, Practical Approach to Care Kit; PICOT, participant/population, intervention/indicator, control/alternative intervention, outcome and timeframe format.

Our initial approach, in collaboration with a Cochrane South Africa team, was to select key recommendations for each topic. We embarked on using the PICOT (participant/population, intervention/indicator, control/alternative intervention, outcome and timeframe format)^26^ and GRADE^27^ approach to determine the evidence for these recommendations. This process proved onerous and was impossible to scale across thousands of PACK guide recommendations. In addition, these approaches are not suited for all types of clinical recommendation,^28^ and our process inevitably focused on recommendations for which there was robust evidence (eg, inhaled corticosteroids to prevent asthma exacerbations). Limiting the search to key recommendations left the rest without a transparent record for inclusion and no streamlined way of updating. We also wanted to include clear, pragmatic recommendations for scenarios where evidence was scanty in LMIC contexts (eg, for tuberculosis diagnosis in pregnancy).

A search for groups conducting comprehensive evidence-horizon scanning led to a 5-year partnership with British Medical Journal (BMJ) Publishing Group. The Group’s 60-person Knowledge Centre produces the evidence-based point-of-care tool, BMJ Best Practice.^29^ Best Practice comprises over 1000 monographs, which are revised at least annually with systematic searching of the evidence horizon and robust editorial and peer-review processes.^30^ The partnership allowed us to check each of the 3689 PACK Adult Western Cape 2015 guide^31^ recommendations against Best Practice guidance. We also linked PACK recommendations to WHO’s clinical and policy guidelines and Essential Medicines List (EML).^32^ Discrepancies between these two sources were largely due to three scenarios: Best Practice medication recommendation not included in WHO EML, Best Practice recommendation omitting an LMIC differential diagnosis (eg, tuberculosis in lymphadenopathy) and WHO guidance containing out-of-date recommendations. We resolved these, as well as gaps in low-resource scenario evidence, as an editorial team on a case-by-case basis, consulting sentinel international guidelines, primary studies and other online point-of-care medical resources like UpToDate.^33^ We adjusted PACK recommendations accordingly to be evidence-based, aligned wherever possible to WHO guidance and pragmatic for a low-resource setting.

The PACK guide is available to localiser and end-user (primary care nurse, doctor, clinical officer or community health extension worker) as a hard copy manual or soft copy interactive pdf.

**Figure 1:**
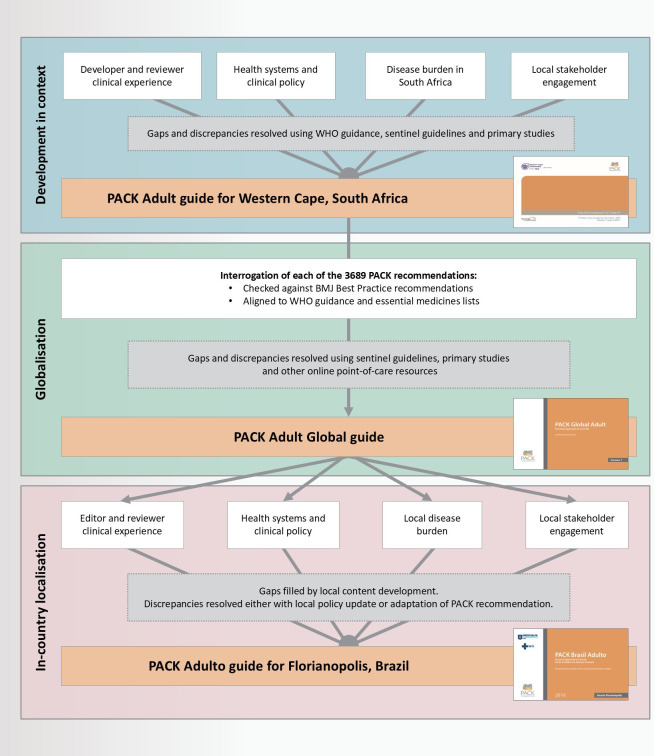
Development of the PACK guide. BMJ, British Medical Journal; PACK, Practical Approach to Care Kit.

PACK guide development and localisation is explained further elsewhere^15 34^ and summarised in figure 1.

### The PACK reference repository

PACK guide content is held in a database that stores the evidence for each of its recommendations. This database is currently accessed only by the PACK editorial team; PACK localisers receive a report derived from the database for the relevant guide.

Development was iterative as design and functionality needs emerged: be user-friendly, allow multiple-user access, facilitate editorial processes and be robust enough for both data volume and recommendation granularity. Additional requirements included for data provenance, source alignment tracking and storage of multiple guide versions.

**Figure 2:**
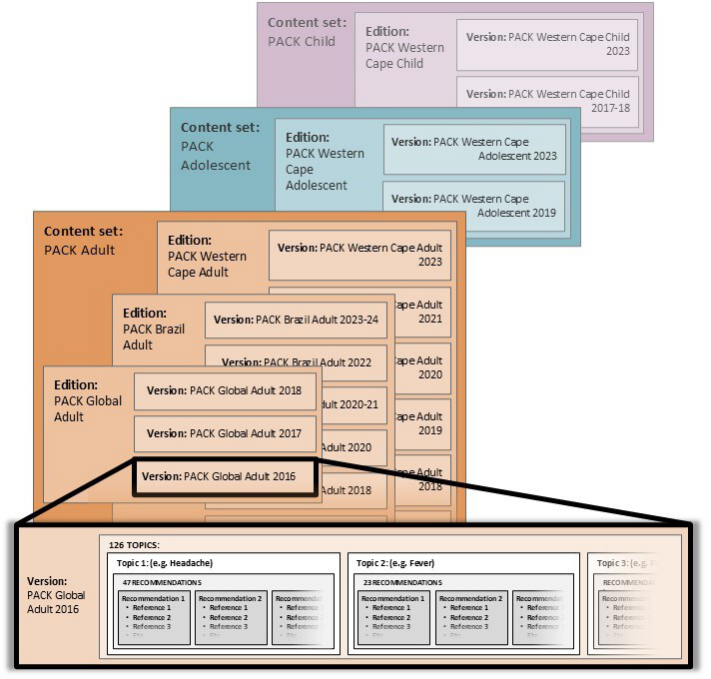
Hierarchical structure of a PACK content set. PACK, practical approach to care kit.

The database has a bespoke web-based user interface linked to a Microsoft Structured Query Language server database. Its functional layout is displayed in figure 2.

This database structure allows for age-limited content sets, with each guide (PACK Adult, PACK Adolescent and PACK Child) stored separately. Each content set contains various editions (territory and year), with PACK Adult Global the template for localisations like PACK Brasil Adulto.^9^ The existing version (eg, PACK Global 2017) serves as the starting point for an update (eg, PACK Global 2018). Within each content set, clinical topics (mean=126; eg, “Weight loss”, “HIV: diagnosis”) are housed. Each version of topics within a content set holds related recommendations per topic (median=26, range 1–79; eg, Weight loss topic recommendation: “Investigate unintentional weight loss of ≥5% of body weight in last 6 months.”; HIV topic recommendation: “Assess for tuberculosis at every visit”). Recommendations are categorised into four activities: screening (n=413, 12%), diagnosis (n=961, 28%), pharmacological management (n=1026, 30%) and non-pharmacological management (n=1095, 31%). Each recommendation lists related evidence sources (median=3, IQR=2–4, with 90% from either WHO or BMJ-Best Practice) and, where both WHO and BMJ sources are included, whether they agree. Online supplemental file 1 itemises number of recommendations per topic and references per recommendation in the PACK Adult Global 2017 guide.

These data points are linked to a password-secured user profile when entered or updated on the database, ensuring accountable tracking of changes.^35^ The database supports content curation with buttons for editorial sign-off and fields for deliberation between evidence researcher, medical writer and editor about recommendation and reference details.

Each recommendation can include tips for a PACK localiser. These include pragmatic guidance given local burden of disease and medication resistance patterns, treatment thresholds, alternatives for unavailable tests or medications and guidance around priority-setting where resources are limited.

Online supplemental file 2 provides an example of a PACK Adult Global 2017 recommendation, along with its references and displaying the database interface.

The database produces the following reports:

- Evidence and Decision Support Documents: generated per topic for localiser to consult recommendation references.
- Number of topics, recommendations and references.
- Revision Report: provides a view of data for revised recommendations.
- Summary of Changes Report: compares recommendation amendments with that of the previous version. Published alongside new editions to flag updates.
- Weblink Search Report: allows editors to search for references with text in their web links and access the direct link to a reference source.

### Maintaining and updating PACK and its reference repository

We published two updates to the PACK Global Adult guide since its 2016 release: in 2017 and 2018.

That 93% of PACK recommendations linked to Best Practice allowed us to develop streamlined referencing and updating systems for much of the content. We worked with the Knowledge Centre to screen weekly Best Practice changes. This allowed us to consider a small set of documents and leverage the comprehensive and robust Best Practice screening and update processes.

We also developed a system to scan for new and updated WHO publications. We determined a list of 12 priority topics (communicable diseases, non-communicable diseases and mental health, as well as the WHO EML) and checked weekly for updates to the WHO Health topics webpage.^36^ Every month, we searched the WHO Institutional Repository for Information Sharing^37^ for new or revised WHO guidance pertaining to PACK content.

We logged suggested changes to the PACK guide on a pdf version of the current edition. Unless urgent (such as for revised medication dosages or an infectious disease outbreak), flagged changes were considered in the PACK guide annual review cycle. This was accompanied by input of the updated clinical recommendation and reference in the appropriate topic section of the database for the new version.

Update processes also drew on refinements to the PACK guide during localisations and local updates, incorporating local stakeholder input in the form of end-user feedback and testing of priority content (eg, tuberculosis diagnosis), clinical specialist review and policy-maker sign-off. This included co-development of new content to respond to local disease burden (eg, dengue fever) and health system priorities (tobacco cessation), as well as usability enhancements drawn from end-user feedback (adding body mass index-based cardiovascular disease risk charts for areas where cholesterol testing is limited).

In 2022, the PACK Adult content was adapted for use by the WHO Integrated Clinical Services Department. This provided an opportunity for an overdue, systematic update, ensuring alignment to latest WHO guidance and adding new content (such as neglected tropical diseases and hepatitis B and C). The database structure allowed us to work systematically through each topic, recommendations and references, producing each topic along with its Evidence and Decision Support document. We added localiser’s notes to consider disease burden (eg, prevalence of soil-transmitted helminth infestation in anaemia management) and resource availability (eg, of molecular WHO-recommended rapid TB diagnostics^38^). In total, 126 topics were covered, including 3495 recommendations. As of 2025, this remains unpublished.

### Tackling the challenges of maintaining a comprehensive content set for LMIC primary care

#### Comprehensive scope of PACK content remains overwhelming

A big challenge to regular PACK guide updates was that reviewing new (often lengthy) WHO publications proved too time-consuming for our small team. The comprehensive scope of primary care necessitated the review of over 24 000 pages of 236 WHO guidelines for the 2022 PACK update.

Advances in artificial intelligence (AI) provide potential to support LMIC public health initiatives.^39^ We are building a bespoke AI-enabled knowledge library of policy documents and other relevant sources. We aim to use supervised large language models (LLMs) to assist with data interrogation and clinical recommendation extraction of these sources with a human-in-command approach,^40^ thus diminishing the load for PACK editors. Careful source curation to include guidance only from WHO, sentinel guidelines and LMIC clinical policies may help to level the data bias inherent in generic LLMs trained on content from the internet and Global North publications.

While AI offers several solutions to our model’s limitations, it also brings challenges. Knowledge library development and maintenance costs are not inconsiderable. The risks of AI-generated clinical content being, for example, biased, hallucinated or lacking transparency can have severe repercussions on patient care and outcomes.^41^ We plan to mitigate these risks by limiting AI use to defined steps in the workflow of document curation, summarisation and query extraction, with careful sequencing and checking by the editorial team. In addition, our AI-enabled platform still requires full evaluation and ultimately registration with a regulatory body to ensure its rigorous application.

#### Funding is limited for ‘global’, disease-agnostic content

Donor funds tend to be bound by disease or research project and rarely have a comprehensive, multicondition focus. Contracts with government health departments, technical support partners and research donors to fund PACK guide localisations or local updates have not supported maintaining the Global guide. These constraints have prevented annual publications since 2019.

We are exploring with PACK country partners a social franchise model of funding for PACK localisations, updates and implementations that could cover the cost of maintaining the PACK generic content set. This model shows potential as a strategy to expand, improve and sustain service delivery.^42^

We recently formed a Global-South-led PACK cross-country research collaboration to create a platform for sharing PACK implementation experiences and conducting research on its impact. We hope that profiling PACK’s contributions to health system strengthening will make a compelling case for renewed funding. This is timely given current reflection on funding models that prioritised Global-North-led, disease-focused, short-term impacts and proved vulnerable to changing global politics.

#### Local PACK guide updates are time-pressured

Localisations and local updates of the PACK guide often are time-pressured both by rapid churn of evidence (especially around HIV and tuberculosis management), as well as by short-term government contracts. This leaves little time to populate the reference repository simultaneously—a missed opportunity to provide a transparent evidence base for local versions of PACK and systematically track policy shifts over time and across countries. We are exploring whether using the AI-driven knowledge library will expedite the document review process, thus allowing time to populate the reference repository for each local edition.

#### Finalising comprehensive guidance for publication is a complex process

As new guidance emerges frequently, efforts to get a comprehensive content set finalised can be waylaid by experts and directorates who defer publication until latest guidance is incorporated. We attempt to mitigate this through clear annual cycles and deadlines, after which new guidance is held over for the next review cycle. Annual PACK publication events are needed for hard copy versions and help boost implementation efforts but run the risk of presenting out-of-date content. Digital editions and ‘living guidelines’ offer a solution^43^; however, considerable resources are required to maintain a living guideline (especially one with a broad clinical focus).^44^ There is little published around facilitators and barriers of living guideline use, particularly in LMICs^45^—likely factors include compromised access due to energy poverty, connectivity issues, digital literacy and data costs. In South Africa, a preference for hard copies of PACK remains, outnumbering downloaded versions by a factor of 10. Presenting a living PACK guide via low-bandwidth platforms or offline might make it more accessible to those with unreliable internet connectivity.

#### Streamlining content creation processes is challenging

Creation and adjustment of PACK guide topics is cumbersome as our clinical editorial and referencing processes remain separate steps from design and layout. This is a factor both of the content’s design-intensive, algorithmic nature and database limitations to support direct publication to PACK’s visual layout. Costs and time to develop a bespoke content management system are barriers shared in better-resourced settings.^46^ Advances in computing and software capabilities might allow integration of graphical tools with the database and support layout processes.

## Conclusion

Building a reference repository to underpin PACK guide content had several advantages—streamlining editorial processes, making guide development more transparent, facilitating localisation decisions and providing a systematic updating mechanism. Drawing on the BMJ partnership and regularly scanning WHO sources saw us leveraging two better-resourced evidence synthesis groups to create, with a small team, up-to-date comprehensive guidance for LMIC primary care health workers.

Our work recognises the poor representation of Global South guidance and policies in guideline repositories and attempts to address limited Global South collaboration to share guideline localisation efforts for LMIC settings.

It is, however, hampered by funding and resource constraints. Leveraging the potential of generative AI and modern database software could augment our approach and offer a solution to combating information inequalities and access disparities in the evidence-based clinical resource space.

## Supplementary material

10.1136/bmjgh-2023-013814 online supplemental file 1

10.1136/bmjgh-2023-013814 online supplemental file 2

## Data Availability

Data sharing not applicable as no datasets generated and/or analysed for this study.

## References

[1] Alper BS, Hand JA, Elliott SG (2004). How much effort is needed to keep up with the literature relevant for primary care?. J Med Libr Assoc.

[2] PubMed website. https://pubmed.ncbi.nlm.nih.gov/.

[3] Kwag KH, González-Lorenzo M, Banzi R (2016). Providing Doctors With High-Quality Information: An Updated Evaluation of Web-Based Point-of-Care Information Summaries. J Med Internet Res.

[4] Hudspeth J, Morse M (2017). Health Information and Global Health Inequity: Point-of-Care Knowledge Systems as a Foundation for Progress. J Gen Intern Med.

[5] Schünemann HJ, Wiercioch W, Brozek J (2017). GRADE Evidence to Decision (EtD) frameworks for adoption, adaptation, and de novo development of trustworthy recommendations: GRADE-ADOLOPMENT. J Clin Epidemiol.

[6] Maaløe N, Ørtved AMR, Sørensen JB (2021). The injustice of unfit clinical practice guidelines in low-resource realities. Lancet Glob Health.

[7] Adult Primary Care (APC) Clinical Tool (2023). National department of health of South Africa. https://knowledgehub.health.gov.za/elibrary/adult-primary-care-apc-clinical-tool-2023.

[8] Tsima BM, Setlhare V, Nkomazana O (2016). Developing the Botswana Primary Care Guideline: an integrated, symptom-based primary care guideline for the adult patient in a resource-limited setting. J Multidiscip Healthc.

[9] Wattrus C, Zepeda J, Cornick RV (2018). Using a mentorship model to localise the Practical Approach to Care Kit (PACK): from South Africa to Brazil. *BMJ Glob Health*.

[10] Feyissa YM, Hanlon C, Emyu S (2019). Using a mentorship model to localise the Practical Approach to Care Kit (PACK): from South Africa to Ethiopia. BMJ Glob Health.

[11] Awotiwon A, Sword C, Eastman T (2018). Using a mentorship model to localise the Practical Approach to Care Kit (PACK): from South Africa to Nigeria. BMJ Glob Health.

[12] Fairall L, Cornick R, Bateman E (2018). Empowering frontline providers to deliver universal primary healthcare using the Practical Approach to Care Kit. BMJ.

[13] Simelane ML, Georgeu-Pepper D, Ras C-J (2018). The Practical Approach to Care Kit (PACK) training programme: scaling up and sustaining support for health workers to improve primary care. BMJ Glob Health.

[14] Ras C-J From face-to-face to online – learnings from new training models to support health system strengthening initiatives in low resource primary care settings. for publication as part of this pack collection in.

[15] Cornick R, Wattrus C, Eastman T (2018). Crossing borders: the PACK experience of spreading a complex health system intervention across low-income and middle-income countries. BMJ Glob Health.

[16] Bachmann MO, Bateman ED, Stelmach R (2019). Effects of PACK guide training on the management of asthma and chronic obstructive pulmonary disease by primary care clinicians: a pragmatic cluster randomised controlled trial in Florianópolis, Brazil. BMJ Glob Health.

[17] Shekar S, Bachmann MO, Bateman ED (2024). Effects of PACK training on the management of asthma and chronic obstructive pulmonary disease by primary care clinicians during 2 years of implementation in Florianópolis, Brazil: extended follow-up after a pragmatic cluster randomised controlled trial with a stepped-wedge design. BMJ Glob Health.

[18] Zonta R, Zaros Galana M, Zepeda J (2024). Supporting a rapid primary care response to emergent communicable disease threats with PACK (Practical Approach to Care Kit) in Florianópolis, Brazil. BMJ Glob Health.

[19] Cornick RV, Petersen I, Levitt NS (2024). Clinically sound and person centred: streamlining clinical decision support guidance for multiple long-term condition care. BMJ Glob Health.

[20] Kibret AG, Belete WM, Hanlon C (2024). Ethiopian primary healthcare clinical guidelines 5 years on-processes and lessons learnt from scaling up a primary healthcare initiative. BMJ Glob Health.

[21] Picken S, Hannington J, Fairall L (2018). PACK Child: the development of a practical guide to extend the scope of integrated primary care for children and young adolescents. *BMJ Glob Health*.

[22] Curran R, Murdoch J, Bachmann M (2021). Addressing the quality of paediatric primary care: health worker and caregiver perspectives from a process evaluation of PACK child, a health systems intervention in South Africa. BMC Pediatr.

[23] Murdoch J, Curran R, Cornick R (2020). Addressing the quality and scope of paediatric primary care in South Africa: evaluating contextual impacts of the introduction of the Practical Approach to Care Kit for children (PACK Child). BMC Health Serv Res.

[24] Mash B, Fairall L, Adejayan O (2012). A morbidity survey of South African primary care. PLoS One.

[25] Schünemann HJ, Wiercioch W, Etxeandia I (2014). Guidelines 2.0: systematic development of a comprehensive checklist for a successful guideline enterprise. CMAJ.

[26] Gallagher Ford L, Melnyk BM (2019). The Underappreciated and Misunderstood PICOT Question: A Critical Step in the EBP Process. Worldviews Evid Based Nurs.

[27] Brozek JL, Akl EA, Alonso-Coello P (2009). Grading quality of evidence and strength of recommendations in clinical practice guidelines. Part 1 of 3. An overview of the GRADE approach and grading quality of evidence about interventions. Allergy.

[28] Huang X, Lin J, Demner-Fushman D (2006). Evaluation of PICO as a knowledge representation for clinical questions. AMIA Annu Symp Proc.

[29] BMJ best practice website. https://bestpractice.bmj.com/info/.

[30] Best Practice website Regularly updated, BMJ best practice draws on the latest evidence-based research. https://bestpractice.bmj.com/info/benefits-features/evidence-based/.

[31] Western Cape Department of Health (2015). Practical approach to care kit (pack) primary care guide for the adult.

[32] WHO model lists of essential medicines. https://www.who.int/groups/expert-committee-on-selection-and-use-of-essential-medicines/essential-medicines-lists.

[33] UpToDate website. https://www.uptodate.com/contents/search.

[34] Cornick R, Picken S, Wattrus C (2018). The Practical Approach to Care Kit (PACK) guide: developing a clinical decision support tool to simplify, standardise and strengthen primary healthcare delivery. BMJ Glob Health.

[35] Flott K, Maguire J, Phillips N (2021). Digital safety: the next frontier for patient safety. *Future Healthc J*.

[36] World health organisation health topics webpage. https://www.who.int/health-topics.

[37] WHO IRIS institutional repository for information sharing. https://apps.who.int/iris/.

[38] (2022). Manual for selection of molecular who-recommended rapid diagnostic tests for detection of tuberculosis and drug-resistant tuberculosis. https://www.who.int/publications/i/item/9789240042575.

[39] Weeks WB, Taliesin B, Lavista JM (2023). Using Artificial Intelligence to Advance Public Health. Int J Public Health.

[40] Perlis RH, Fihn SD (2023). Evaluating the Application of Large Language Models in Clinical Research Contexts. *JAMA Netw Open*.

[41] Chustecki M (2024). Benefits and Risks of AI in Health Care: Narrative Review. Interact J Med Res.

[42] Rahman MH, Perkins JE, Usmani NG (2025). Social franchising in healthcare: a systematic review and narrative synthesis of implementation and outcomes. BMJ Glob Health.

[43] Akl EA, Meerpohl JJ, Elliott J (2017). Living systematic reviews: 4. Living guideline recommendations. J Clin Epidemiol.

[44] Cheyne S, Fraile Navarro D, Hill K (2023). Australian Living Evidence Consortium Methods and Processes Working Group and Collaborators. Methods for living guidelines: early guidance based on practical experience. Paper 1: Introduction. J Clin Epidemiol.

[45] Meteku BT, Quigley M, Turner T (2024). Barriers to and facilitators of living guidelines use in low-income and middle-income countries: a scoping review. BMJ Open.

[46] Reddy S, Herring S, Gray A (2017). Identifying an appropriate Content Management System to develop Clinical Practice Guidelines: A perspective. Health Informatics J.

