# Supplementary material for: Addressing health information inequities: making evidence-based clinical content more accessible in low- and middle-income primary care
Source: BMJ Glob Health. 2025 Dec 19;9(Suppl 3):e013814. doi: 10.1136/bmjgh-2023-013814 (PMC12917359; doi:10.1136/bmjgh-2023-013814)
Supplement: online supplemental file 1 [file bmjgh-9-Suppl_3-s001.docx]

**Supplemental file 1. Recommendations per topic and references per recommendation in the PACK Adult Global 2017 guide**

| **Topic Classification** | **Topic** | **Recommendations** | **References** |
| --- | --- | --- | --- |
| **Symptom** | **61** | **1353** | **5540** |
|  | Abdominal pain (no diarrhoea) | 31 | 151 |
|  | Abnormal thoughts or behaviour | 25 | 167 |
|  | Abnormal vaginal bleeding | 29 | 92 |
|  | Aggressive/disruptive patient | 17 | 78 |
|  | Anal symptoms | 7 | 39 |
|  | Arm symptoms | 10 | 51 |
|  | Back pain | 23 | 91 |
|  | Bites and stings | 21 | 76 |
|  | Body/general pain | 13 | 46 |
|  | Breast symptoms | 27 | 85 |
|  | Burn/s | 12 | 63 |
|  | Cervical screening | 8 | 20 |
|  | Changes in skin colour | 41 | 132 |
|  | Chest pain | 33 | 104 |
|  | Collapse/falls | 16 | 93 |
|  | Constipation | 11 | 53 |
|  | Cough or difficulty breathing | 32 | 125 |
|  | Diarrhoea | 19 | 70 |
|  | Difficulty sleeping | 15 | 74 |
|  | Dizziness | 22 | 110 |
|  | Ear/ hearing symptoms | 19 | 82 |
|  | Exposed to infectious fluid: post-exposure prophylaxis | 19 | 123 |
|  | Eye/vision symptoms | 27 | 113 |
|  | Face symptoms | 30 | 124 |
|  | Fever | 24 | 139 |
|  | Foot symptoms | 28 | 90 |
|  | Generalised itchy rash | 27 | 104 |
|  | Generalised non-itchy rash | 23 | 72 |
|  | Genital symptoms | 20 | 94 |
|  | Genital symptoms in a man | 19 | 69 |
|  | Genital ulcer | 15 | 68 |
|  | Headache | 49 | 161 |
|  | Itch with no rash | 12 | 37 |
|  | Joint symptoms | 10 | 45 |
|  | Leg symptoms | 23 | 64 |
|  | Localised itchy rash | 18 | 60 |
|  | Lump/s in neck, axilla or groin | 22 | 64 |
|  | Mouth and throat symptoms | 39 | 130 |
|  | Nail symptoms | 22 | 68 |
|  | Nausea or vomiting | 28 | 107 |
|  | Neck pain | 7 | 53 |
|  | Nose symptoms | 23 | 110 |
|  | Other genital symptoms | 17 | 50 |
|  | Painful skin | 22 | 92 |
|  | Positive syphilis result | 15 | 62 |
|  | Seizures/fits | 26 | 157 |
|  | Self-harm or suicide | 24 | 95 |
|  | Sexual problems | 34 | 125 |
|  | Skin lump/s | 32 | 106 |
|  | Skin symptoms | 16 | 73 |
|  | Stressed or miserable patient | 21 | 87 |
|  | The emergency patient | 18 | 82 |
|  | The injured patient | 40 | 163 |
|  | The unconscious patient | 19 | 120 |
|  | Traumatised/abused patient | 19 | 84 |
|  | Ulcers and crusts | 36 | 120 |
|  | Urinary symptoms | 26 | 89 |
|  | Vaginal discharge | 23 | 108 |
|  | Weakness or tiredness | 25 | 137 |
|  | Weight loss | 19 | 65 |
|  | Wheeze/tight chest | 5 | 28 |
| **Chronic condition** | **36** | **1089** | **4288** |
|  | Admit the mentally ill patient | 8 | 11 |
|  | Alcohol/drug use | 23 | 124 |
|  | Asthma and COPD: diagnosis | 9 | 34 |
|  | Asthma: routine care | 18 | 83 |
|  | Cardiovascular disease (CVD) risk: diagnosis | 12 | 60 |
|  | Cardiovascular disease (CVD) risk: routine care | 17 | 104 |
|  | Chronic arthritis: diagnosis and routine care | 21 | 62 |
|  | Chronic obstructive pulmonary disease (COPD): routine care | 22 | 67 |
|  | Dementia: diagnosis and routine care | 26 | 103 |
|  | Depression and/or anxiety: routine care | 36 | 172 |
|  | Depression: diagnosis | 15 | 91 |
|  | Diabetes: diagnosis | 17 | 66 |
|  | Diabetes: routine care | 42 | 241 |
|  | Drug-sensitive (DS) TB: routine care | 50 | 123 |
|  | Epilepsy: routine care | 33 | 123 |
|  | Fibromyalgia: diagnosis and routine care | 22 | 63 |
|  | Gout: diagnosis and routine care | 22 | 91 |
|  | Heart failure: routine care | 41 | 155 |
|  | HIV: diagnosis | 22 | 64 |
|  | HIV: routine care | 79 | 308 |
|  | Hypertension: Diagnosis | 13 | 72 |
|  | Hypertension: routine care | 38 | 224 |
|  | Ischaemic heart disease (IHD): initial assessment | 13 | 54 |
|  | Ischaemic heart disease (IHD): routine care | 27 | 63 |
|  | Life-limiting illness: routine palliative care | 32 | 99 |
|  | Menopause | 25 | 73 |
|  | Peripheral vascular disease (PVD): diagnosis and routine care | 25 | 59 |
|  | Routine antenatal care | 65 | 327 |
|  | Routine postnatal care | 58 | 240 |
|  | Schizophrenia: diagnosis and routine care | 47 | 166 |
|  | Start or change ART in the patient with HIV | 58 | 234 |
|  | Stroke: diagnosis and routine care | 29 | 131 |
|  | The pregnant patient | 59 | 211 |
|  | Tobacco smoking | 38 | 98 |
|  | Tuberculosis (TB): diagnosis | 22 | 74 |
|  | Using a peak expiratory flow meter | 5 | 18 |
| **Other** | **8** | **189** | **704** |
|  | Address the patient's general health | 34 | 165 |
|  | Contraception | 69 | 252 |
|  | Prescribe rationally | 15 | 40 |
|  | Prevent mother-to-child transmission (PMTCT) of HIV | 18 | 49 |
|  | Protect yourself from occupational infection | 20 | 66 |
|  | Protect yourself from occupational stress | 15 | 37 |
|  | Review the patient on post-exposure prophylaxis | 17 | 89 |
|  | Support the patient to make a change | 1 | 6 |
| **Total** | **105** | **2631** | **10532** |
