# Supplementary material for: Addressing health information inequities: making evidence-based clinical content more accessible in low- and middle-income primary care
Source: BMJ Glob Health. 2025 Dec 19;9(Suppl 3):e013814. doi: 10.1136/bmjgh-2023-013814 (PMC12917359; doi:10.1136/bmjgh-2023-013814)

**Supplemental file 2. Example of a recommendation and corresponding references in PACK database**

Content set and edition: PACK Adult Global 2017

Topic: Asthma: routine care


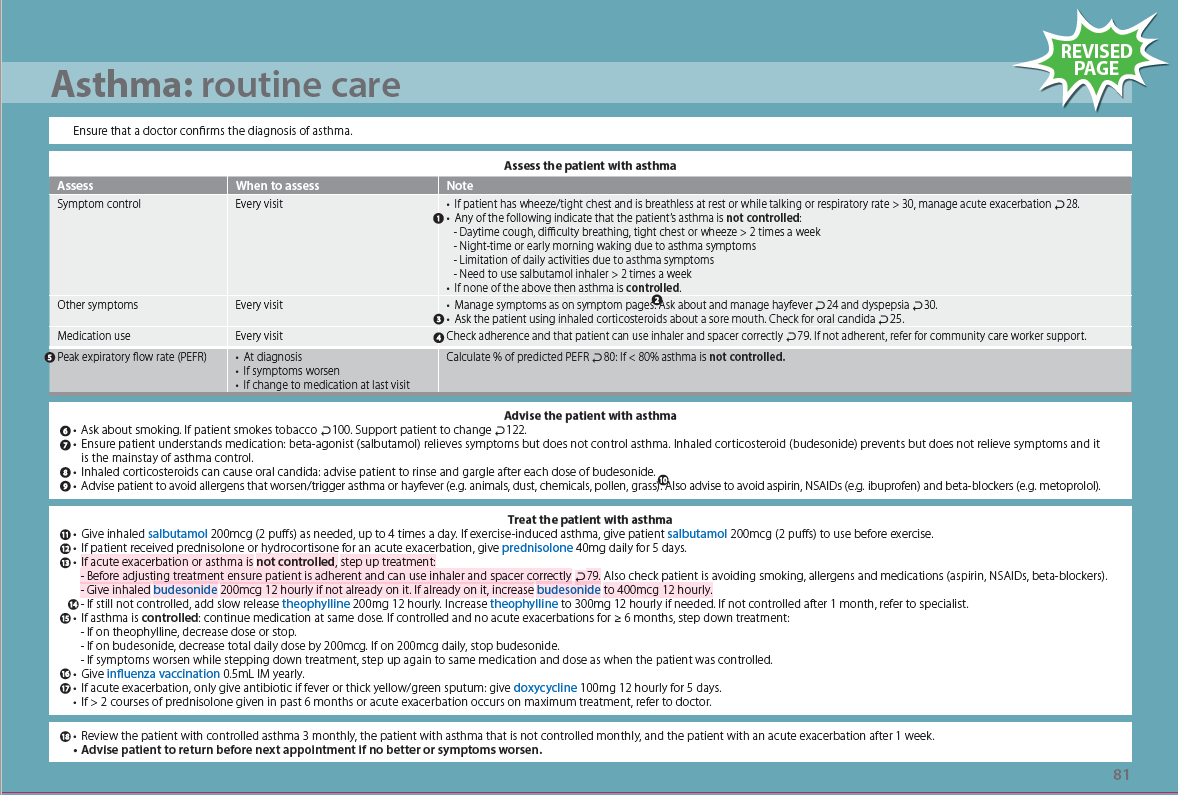


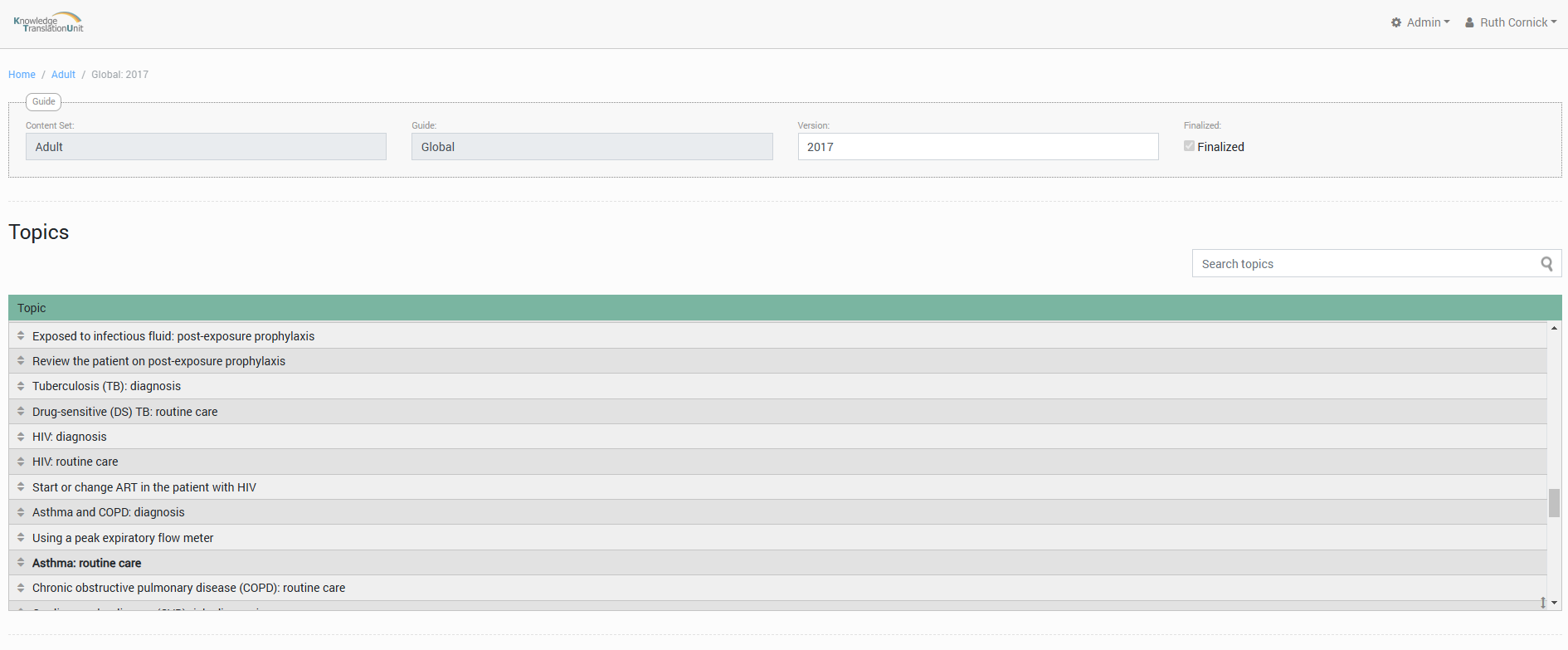


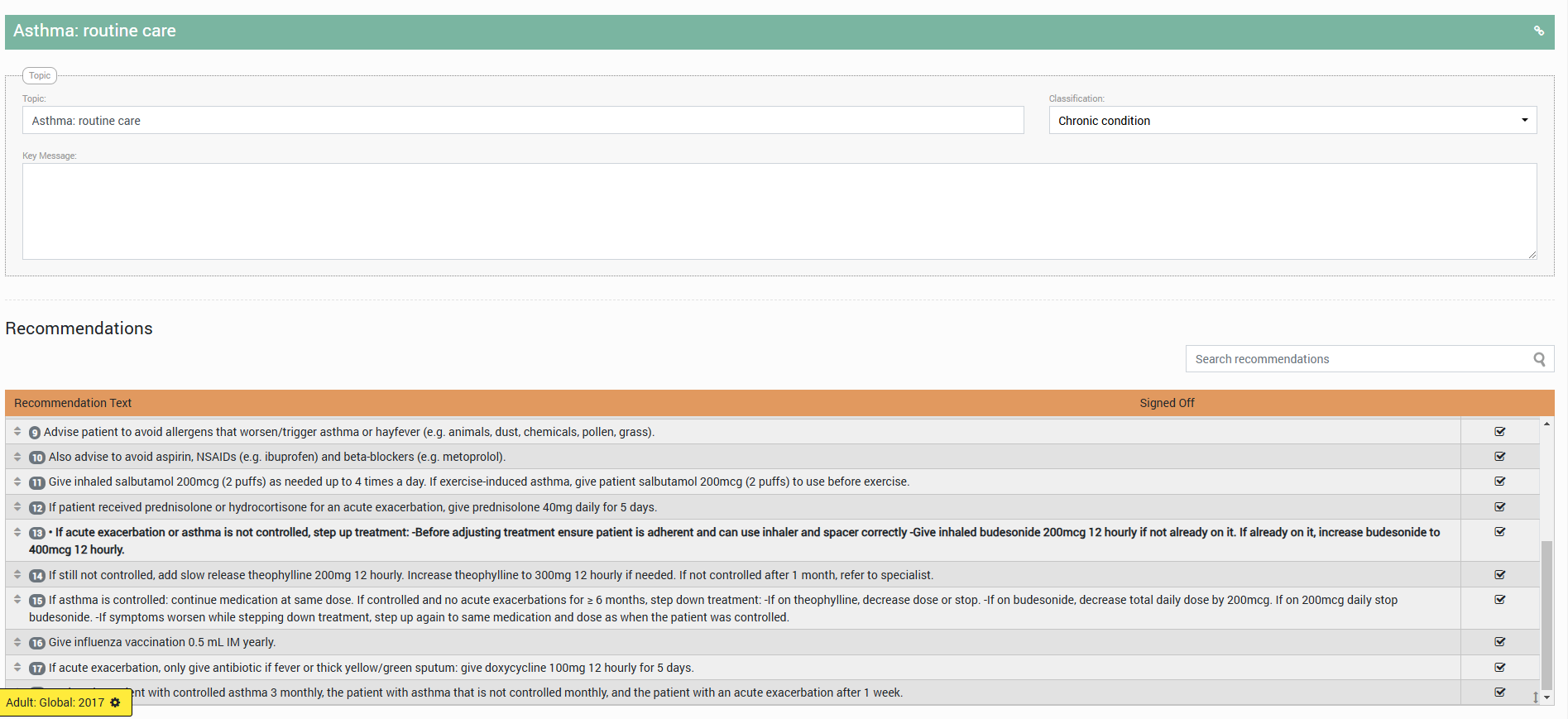


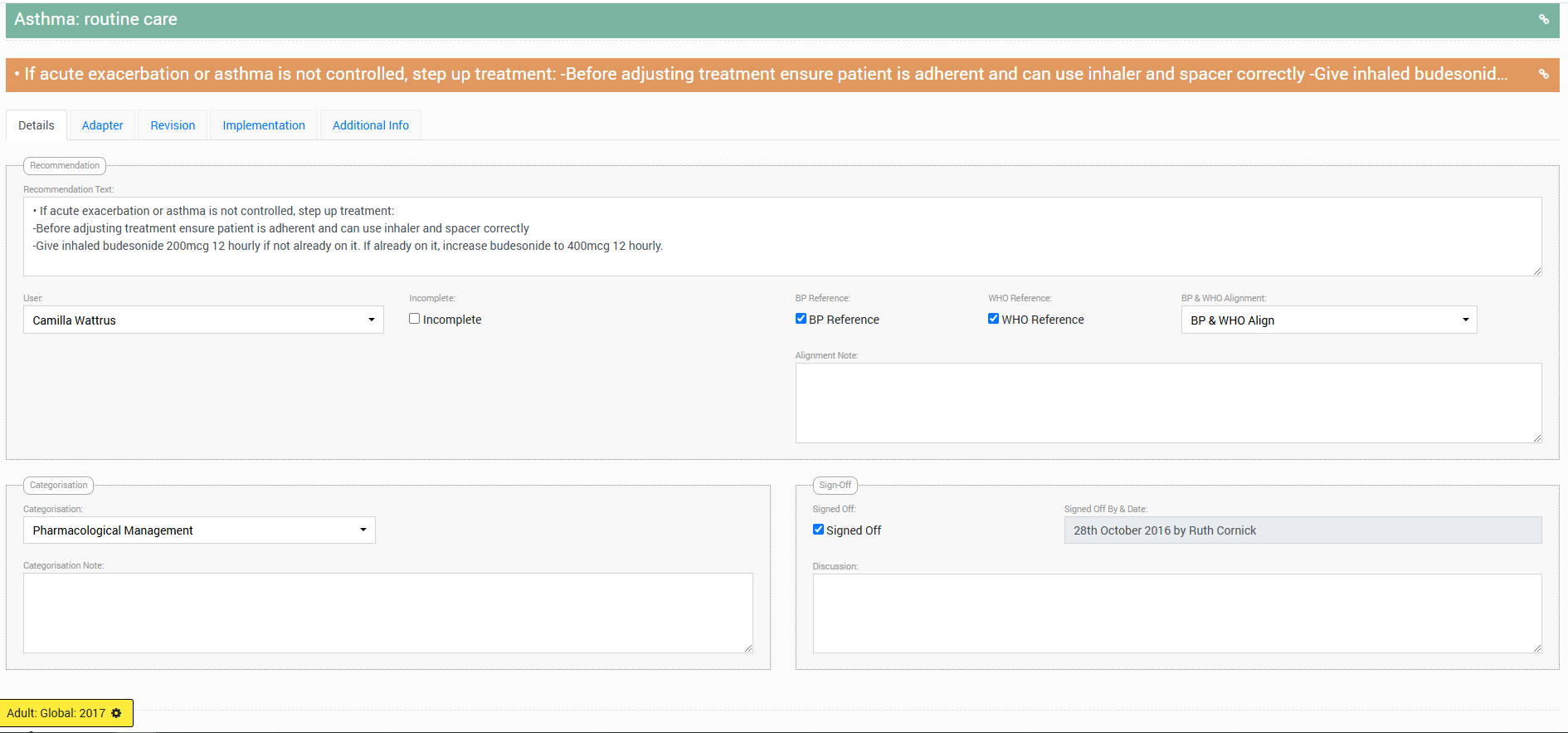


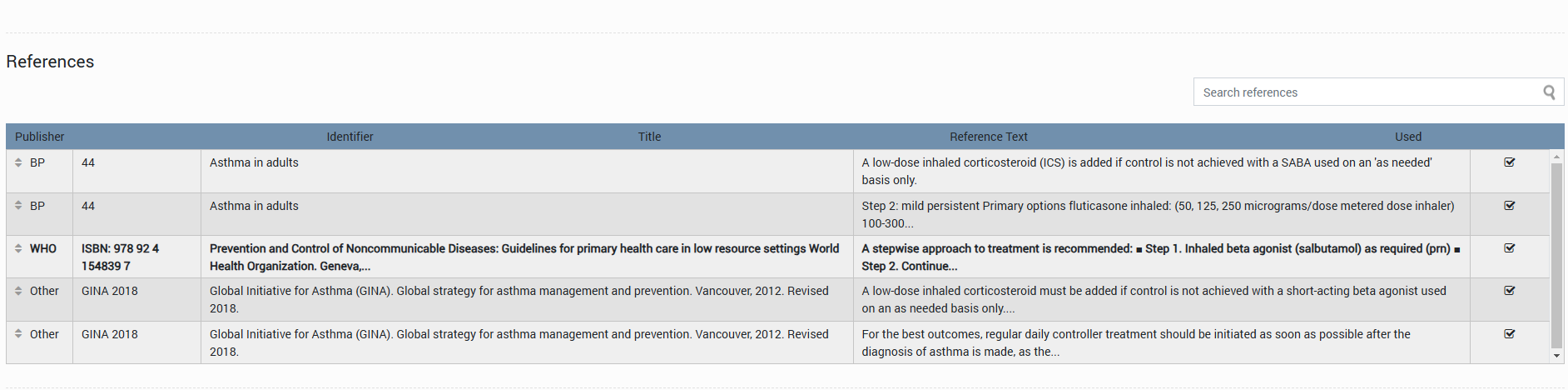


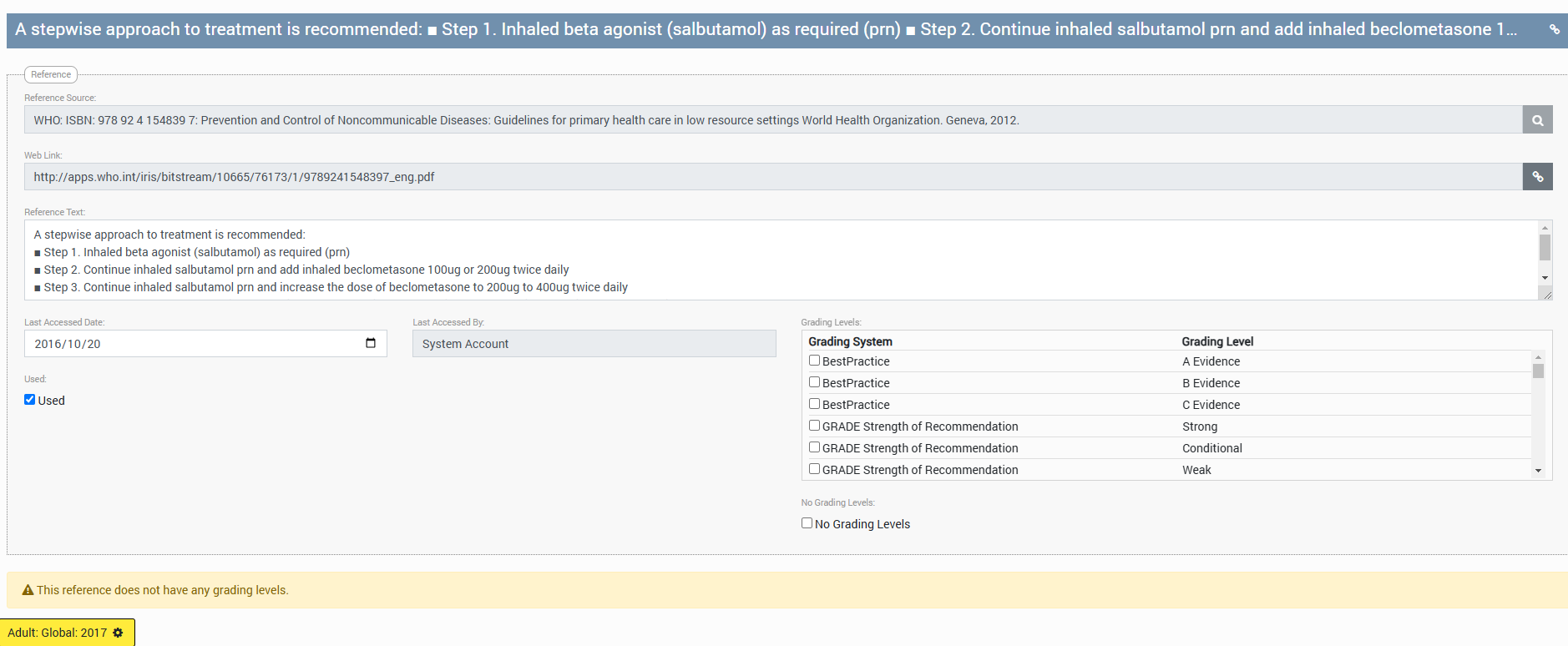

Supplement: online supplemental file 2 [file bmjgh-9-Suppl_3-s002.docx]
